# Vitamin D in healthy Tunisian population: Preliminary results

**DOI:** 10.5937/jomb0-30247

**Published:** 2022-04-08

**Authors:** Afef Bahlous, Asma Krir, Mehdi Mrad, Mouna Bouksila, Safa Kalai, Osman Kilani, Kateb Elhem Cheour El, Hela Sahli, Nizar Laadhari

**Affiliations:** 1 University of Tunis-El Manar, Pasteur Institute of Tunis, Laboratory of Clinical Biochemistry and Hormonology, Tunisia; 2 University of Tunis-El Manar, La Rabta Hospital, Rheumatology Department, Immuno-Rheumatology Research Laboratory, Tunisia; 3 University of Tunis-El Manar, La Rabta Hospital, Medicine School of Tunis, Rheumatology Department, Tunisia; 4 University of Tunis-El Manar, Charles Nicolle Hospital, Occupational Pathology and Fitness for Work Service, Tunis, Tunisia

**Keywords:** healthy volunteers, parathyroid hormone, prevalence, Tunisia, vitamin D deficiency, zdravi volonteri, paratireoidni hormon, prevalencija, Tunis, deficit vitamina D

## Abstract

**Background:**

Vitamin D deficiency is one of the most common medical conditions worldwide. In Tunisia, several studies evaluated Vitamin D status, but this was concerning specific populations (pregnant women, obese or diabetic patients and children with asthma). The only study that evaluated Vitamin D status in a healthy Tunisian population was conducted by Meddeb and associeties in 2002. The update of data available, based on the currently recommended limits, is necessary. This study aimed to estimate the prevalence of hypovitaminosis D in a healthy Tunisian population, and correlate the values with potential risk factors.

**Methods:**

It was conducted on 209 Tunisian healthy subjects. Data collected included clinical characteristics and dietary intakes. We measured 25-hydroxyvitamin D (25(OH)D), parathyroid hormone (PTH), glycemia, creatinine, calcium, phosphorus, and alkaline phosphatase concentrations. Hypovitaminosis D was retained for 25(OH)D concentrations <75 nmol/L. Vitamin D deficiency was defined by 25(OH)D concentrations <25 nmol/L.

**Results:**

The prevalence of hypovitaminosis D and vitamin D deficiency were respectively 92.3% and 47.6%. The main factors that were significantly associated with low vitamin D levels in our multivariate analysis were veiling, living in rural areas and sunscreen use. However, sex, age, socioeconomic level, phototype, solar exposure score, smoking and bone mass index, were not statistically associated with hypovitaminosis D. The study of relationship between vitamin D status and serum PTH levels showed a significative and negative correlation (P < 0.005).

**Conclusions:**

Given the high prevalence of vitamin D, an adapted health policy is essential. A widespread vitamin D supplementation and food fortification seems to be necessary in Tunisia.

## Abstract (sr)

**Uvod:**

Deficijencija vitamina D je jedno od najčešćih medicinskih stanja širom sveta. U Tunisu je bilo nekoliko prouča vanja statusa vitamina D, koja su obuhvatila specifične po pulacije (trudnice, gojazne ili dijabetične osobe i decu sa astmom). Jedino je Meddab sa saradnicima 2002. godine proučavao status vitamina D kod zdravih osoba. Da bi se dopunili raspoloživi podaci o stanju vitamina D neophodna su dodatna istraživanja. Ovo izučavanje je imalo za cilj da proceni prevalenciju hipovitaminoze D u zdrave populacije u Tunisu, kao i da se vrednosti procene u odnosu na potencijalne faktore rizika.

**Metode:**

Ispitivano je 209 zdravih osoba u Tunisu. Objedinjeni su podaci kliničkih karakteristika kao i načina ishra ne. Mereni su 25-hidroksivitamin D (25(OH)D), paratireo idni hormon (PTH), glikemija, kreatinin, kalcijum, fosfor i alkal - na fosfataza. Hipovitaminoza 25(OH)D je definisana kao koncentracija < 75 nmol/L, a deficijencija kao koncentracija < 25 nmol/L.

**Rezultati:**

Prevalencija hipovitaminoze D i deficijencija vitamina D iznosile su 92,3% i 47,6%. Glavni faktori koji su bili pridruženi sa nivoima vitamina D u našoj mulitivarijantnoj ana lizi bili su način života, život u ruralnim sredinama i zaštita od sunca. Međutim, pol, starost, socioekonomski status, fototip, izlaga nje suncu i indeks koštane mase nisu bili statistički značajni za hipovitaminozu D. Odnos vitamina D i nivoa PTH je bio u značajno negativnoj korelaciji (P < 0,005).

**Zaključak:**

Značajno je praćenje vitamina D, kao i davanje vitamina D kao suplementa populaciji u Tunisu.

## Introduction

The importance of vitamin D in phosphocalcic metabolism has been clearly established. It helps prevent the risk of rickets, fractures, osteopenia and osteoporosis [1].

The increased interest in this hormone is due to the discovery of the ubiquitous tissue distribution of vitamin D receptors [2]. Its involvement goes beyond bone metabolism to act at different levels of human physiology: preventing cancers [3], cardiovascular protection [4], regulation of the immune system [5]. In many recent studies, vitamin D status has been also correlated with the severity of Corona Virus Disease 2019 [6] [7].

Vitamin D deficiency is considered to be one of the most common medical conditions worldwide [8]. Numerous studies around the world have shown a high prevalence of vitamin D deficiency [1] in different degrees, going up to 100% in certain populations [9].

In Tunisia, several studies have evaluated Vitamin D status, but this has concerned specific populations such as pregnant women, obese or diabetic patients, intensive care unit patients and children with asthma. The only study that evaluated Vitamin D deficiency in a healthy Tunisian population was conducted by Meddeb et al. [10] in 2002. In this study, hypovitaminosis D was defined by serum 25-hydroxyvitamin D concentrations under the limit of 38 nmol/L, which has been considered, since many years, insufficient to ensure optimal effect of vitamin D on health [11]. A wide optimal range for vitamin D is reported, and differences of opinion exist as to the definition of vitamin deficiency. Evidence suggests that low serum vitamin D concentrations are associated with an increase in parathyroid hormone (PTH). Yet, the normal vitamin concentrations could be considered as concentrations below which serum PTH increases. These levels are still a matter of debate. The update of data available about vitamin D status in a healthy Tunisian population based on the currently recommended limits is necessary.

This study aimed to estimate the prevalence of hypovitaminosis D in a healthy Tunisian population, and correlate the values with potential risk factors, and PTH levels.

## Materials and Methods

### Subjects

A transversal descriptive study was conducted during the spring of 2017. A total of 209 volunteer subjects aged between 18 and 65 years were selected from the paramedical staff of the Pasteur Institute of Tunis, their relatives, and the employees followed-up by the occupational doctors of the Charles Nicolle Hospital in Tunis.

Non-inclusion criteria were pregnancy, breast-feeding, renal failure, liver, digestive or endocrine illness, high blood pressure, patients with granulomatosis, mycobacterial infection or neoplasia, intake of medication which could possibly interfere with phosphate and calcium metabolism.

Exclusion criteria were the presence of high blood pressure on somatic examination and the abnormalities in blood test results such as renal failure, glycoregulatory disorders or hyperparathyroidism.

### Clinical Characteristics

The data collected included age, sex, socio-economic level (classified as low, medium or high depending on whether the participant was a worker, middle manager or senior manager), habitat (urban or rural), habits (smoking, veiling, sunscreen use), solar exposure level: evaluated by a simplified score [12], a solar exposure score ≥ 3 reflects a sufficient solar exposure.

Physical examination included determination of subjects' phototypes according to Fitzpatrick classification [13], weight, high, size, calculation of the body mass index, waist size and blood pressure measurement.

### Dietary Interview

A dietary interview based on the frequency of consumption of vitamin D and calcium was conducted to evaluate nutritional intakes. The results were interpreted according to the daily intake recommended by the national health security agency.

### Laboratory Studies

The participants were asked to fast for at least 12 h, whereupon morning fasting venous blood samples were drawn for biochemical analyses.

Routine biochemical measurements (glycemia, creatinine, calcium, phosphorus, proteins and alkaline phosphatase) were realized on Cobas Integra 400® analyzer (Roche Diagnostics, Swiss).

Serum 25(OH)D levels and serum parathyroid hormone levels (PTH) were both measured by Electro chemiluminescence Technology (Cobas e411 analyzer, Germany, Roche Diagnostics®). This method is an immunological test for a quantitative determination of 25(OH)D and PTH concentrations based on a competition principle for 25(OH)D and a sandwich principle for the PTH. The inter-assay coefficient of variation for 25(OH)D and PTH were lower than 11% and 4% respectively.

Serum PTH concentrations between 1.6 and 6.9 pmol/L were considered normal. PTH concentrations above or below these ranges were classified as being high and low, respectively.

Vitamin D concentrations were interpreted according to the standards recommended by the research and information group on osteoporosis (Table 1) [14].

**Table 1:** Recommended 25-hydroxyvitamin D concentrations according to the research and information group on osteoporosis.

	25 (OH) D levels	
UNIT	ng/mL	nmol/L
Hypovitaminosis D		
– Deficiency	< 10	< 25
– Insufficiency	10–29	25–74
Optimal level	≥ 30	≥ 75

The choice of 75 nmol/L as a cutoff value agrees with previous studies that demonstrated secondary hyperparathyroidism, increased bone turnover, and decreased bone density at the hip at 25(OH)D serum concentrations below this level [15] [16].

### Statistical Analysis

All statistical analyses were performed using the IBM SPSS STATISTICS Version 20 (SPSS Inc., Chicago, USA).

The comparison of two means on independent series was carried out by Student's t-test, and in the event of a small number, by the non-parametric test of Mann and Whitney.

The comparison of several means on independent series was carried out by Snedecor's F test of parametric variance analysis, and in the event of a small number, by Kruskall-Wallis' H-test of non-parametric variance analysis.

Pearson's Chi-square test was used for comparing the frequencies on independent series.

Correlations between two quantitative variables have been studied by Pearson's correlation coefficient, and in case of invalidity, by the ranks' correlation coefficient for Spearman.

Risk factors research was carried out by calculating the Odds Ratio (OR).

A multivariate analysis in logistic regression (backward stepwise method) was carried out to identify risk factors independently with the event.

A statistical significance level was fixed at 0.05.

### Ethical considerations

All participants gave informed consent. Participation was voluntary, confidential ant not remunerated. The study protocol was approved by the ethics committee of the Pasteur Institute of Tunis.

## Results

Two hundred and nine subjects were initially recruited. Thirteen were excluded for different reasons: one subject had high blood pressure, eight subjects had glucoregulatory disorders and four subjects had hyperparathyroidism. The study population thus consisted of 196 subjects whose main sociodemographic and biochemical characteristics are summarized in Table 2.

**Table 2:** Sociodemographic and biochemical characteristics of the sample (N=196). SD: Standard deviation, BMI: Body mass index, PTH: Parathormone, 25 (OH)D: 25 hydroxyvitamin D

VARIABLE	VALUE
Mean age ± SD (years)	36.9 ± 6.4
Extreme ages (years)	22–56
Sex (F/M)	103/ 93
Female (%)	52
Habitat (urban/rural)	174/22
Urban habitat (%)	88.8
Socioeconomic level<br>– Low (%)<br>– Medium (%)<br>– High (%)	7.1<br>88.8<br>4.1
Phototype <br>– II<br>– III<br>– IV	6<br>189<br>1
Solar exposure score ≥ 3 (%)	10.2%
Sunscreen use (%)	43.4%
Veiled women (N=103) (%)	35%
Smoking<br>Average pack-year ± SD	18.9%<br>7.6 ± 5.2
Average BMI ± SD (kg/m^2^)<br>– Normal weight (%)<br>– Overweight (%)<br>– Obese (%)	25.91 ± 3.93<br>45<br>41<br>14
Average systolic blood pressure ± SD (mmHg)	109.6 ± 11.7
Average diastolic blood pressure ± SD (mmHg)	69.11 ± 10.45
Vitamin D nutritional intakes<br>– Average intakes ± SD (mg/day)<br>– Insufficient intakes (%)	2.88 ± 1.73<br>89.3
Calcium nutritional intakes<br>– Average intakes ± SD (mg/day)<br>– Insufficient intakes (%)	602.12 ± 26991.3
Mean glycemia level ± SD (mmol/L)	5 ± 0.3
Mean serum creatinine level ± SD (μmol/L)	61.6 ± 12.9
Mean serum calcium level ± SD (mmol/L)	2.46 ± 0.07
Mean serum phosphate level ± SD (mmol/L)	1.06 ± 0.15
Mean serum alkaline phosphatase level ± SD (IU/L)	67.56 ± 20.3
Mean serum PTH level ± SD (pmol/L)	4.6 ± 1.7
Mean serum 25 (OH)D level ± SD (nmol/L)	31.8 ± 25.7

The accumulated prevalence of hypovitaminosis D (25(OH)D < 75 nmol/L) and vitamin D deficiency (25(OH)D < 25 nmol/L) were respectively 92.3% and 47.6. Only 15 subjects had optimal concentrations of serum 25(OH)D.

The prevalence of vitamin D deficiency was significantly higher in women (P <0.001) with an OR of 3.26 in univariate study. Multivariate analysis showed that female was not independently related to the risk of vitamin D deficiency.

Age of subjects with vitamin deficiency was close to those with normal vitamin D levels (36.87 ± 6.61 versus 37.63 ± 3.86 years, P = 0.65). Age was not a risk factor of vitamin D deficiency.

Subjects living in rural areas had a significantly higher prevalence of vitamin D deficiency than subjects living in urban areas (P = 0.006) with an OR of 3.995. Multivariate analysis showed that rural habitats was a risk factor independently related to vitamin D deficiency.

Among veiled women, 77.8% had a vitamin D deficiency, compared to 55.2% among unveiled women. Thus, vitamin D deficiency was significantly higher in veiled women (P=0.024) with an OR of 2.838. Multivariate analysis showed that veiling was a risk factor independently related to vitamin D deficiency. The prevalence of vitamin D deficiency was significantly higher in subject using regularly sunscreen, with an OR of 3.012.

Similarly, regular sunscreen use was considered as a risk factor independently related to vitamin D deficiency by a multivariate analysis.

Concerning influence of dietetics, univariate analysis showed there was no significant difference in serum 25(OH)D concentrations between subjects with optimal and those with insufficient intake of vitamin D (P= 0.269) and calcium (P=0.46).

In our study, socioeconomic level, phototype, solar exposure score, smoking and BMI were not risk factors of vitamin D deficiency: no statistically significant difference was noticed between subjects according to those factors.

The study of relationship between vitamin D status and serum PTH levels showed a significative and negative correlation between concentrations of 25(OH)D and PTH (P<0.005).

These data suggest that individuals with vitamin D deficiency and insufficiency had high PTH concentrations (4.9 and 4.42 pmol/L, respectively), while those with vitamin D concentrations above 75 nmol/L had lower levels of PTH (3.7 pmol/L) (Table 3).

**Table 3:** Parathyroid hormone concentrations according to vitamin D range.

Vitamin D (nmol/L)	PTH (pmol/L)<br>mean ± standard deviation
< 25	4.9 ± 1.9
25–74	4.42 ± 1.38
> 75	3.7 ± 1.1

## Discussion

Vitamin D deficiency is one of the globally alarming health problems [17].

In our study, the prevalence of hypovitaminosis D was high in healthy Tunisian subjects affecting 92.3% of the study population (81.1% had a rate of 25 (OH) D <50 nmol/L and 49.5% had a deficiency in vitamin D with 25 (OH) D level < 25 nmol/L). The mean level of 25 (OH) D levels was 31.8 ± 25.7 nmol/L.

Previous studies in Tunisia revealed various rates of vitamin D deficiency but the populations studied had specific diseases such as obesity in the study of Ben Othman et al. [18] and diabetes in the study of Abdessalem et al. [19] The only evaluation of vitamin D status in a healthy Tunisian population was published in 2005 in the study of Meddeb et al. [10]. For a 38 nmol/L cut-off, the prevalence of hypovitaminosis D was 47.6. This prevalence was lower than in our study. The difference could be explained by many factors. Using different assays for 25(OH)D measurement, and different cut-offs for hypovitaminosis D interpretation may be the main reasons. Actually, a higher cut-off currently adopted may explain this increase in hypovitaminosis D prevalence in Tunisia and worldwide.

Many studies have estimated vitamin D deficiency throughout the world. Closely to our results, high prevalence rates of hypovitaminosis D were reported in other countries. A Moroccan study [20] conducted on healthy women found hypovitaminosis D in 91% of cases. In India, several studies on healthy subjects [9] [21] [22] have shown that more than 90% of the Indian population has hypovitaminosis D. In Africa, despite the significant amount of sun exposure, 92% of subjects from East Africa have a 25(OH)D levels < 50 nmol/L according to the study of SA Skull et al. [23]. In Europe, the prevalence of hypovitaminosis D is different depending on the country, the population studied and the limits of interpretation. Indeed, a North-South gradient was noticed, with a lower prevalence of hypovitaminosis D in the Scandinavian countries, where there is a significant consumption of fatty fish and a fortification of certain foods in vitamin D: 80% of healthy French people had a vitamin D level < 75 nmol/L [24] compared to 53% of Danes [25].

The main factors that were significantly associated with low levels of vitamin D in our multivariate analysis were veiling, living in rural areas and sunscreen use.

In our study, age was not a risk factor of vitamin D deficiency. Studies conducted in East Africa [26], the United Arab Emirates [27], and Germany [28]. did not also find a significant relationship between age and vitamin D status. However, other studies highlighted the negative correlation between age and serum vitamin D levels [24] [29]. The effect of age seems to be related to different factors. Actually, cutaneous production of vitamin D in elderly is reduced compared to young adults: a 70-year-old subject produces four times less vitamin D3 than a 20-year-old subject. Also, the food intake of the elderly is generally less, leading to a lower food intake of vitamin D [30]. The effect of age is more pronounced in postmenopausal women, most probably due to the conversion of 25(OH)D into 1,25-dihydroxyvitamin D (1,25(OH)_2_D) under the effect of estrogen contained in hormonal substitution therapy for menopause [31].

Female gender was not independently related to the risk of vitamin D deficiency in our study. Other studies carried out in European countries such as France [24] or Finland [32] have, also, not found any difference between the two sexes. However, several international [22] [25] [26] and Tunisian [10] [18] [19] studies confirm the predominance of hypovitaminosis D in women. This difference between men and women could be explained mainly by the effect of endogenous estrogens which increase the conversion of 25(OH)D to 1,25(OH)_2_D [31]. It could be also explained by clothing habits in some countries with covered clothes for women [1] [33].

Our multivariate study found that veiling was independently linked to the risk of vitamin D deficiency. These results agree with those of Meddeb and al. that found hypovitaminosis D in 70.5% of veiled women and 48.9% in unveiled women (P = 0.006) [10]. Various studies have already shown that veiled women have a two to five times higher risk of vitamin D deficiency [20] [28]. This deficiency increases proportionally to the extent of covered skin [34].

Although living in rural areas was usually described as a protective factor against hypovitaminosis D [35], the findings of our multivariate study showed that living in rural areas was an independent risk factor associated to vitamin D deficiency. Indeed, in our study, 59% of subjects from rural areas were women. Among rural women, 61% were veiled. We mentioned this disparity between the gender of participants of rural and urban origin and their clothing habits to explain the higher prevalence observed in rural areas in our study, but the multivariate study rejected this hypothesis. Certain parameters des cri bed to influence circulating levels of 25(OH)D, such as altitude [36], number of children [28], or month of birth [37], that have not been considered in our study, may also have affected our results. In addition, des pite of living in rural areas, the participants in our study were city workers, thus their lifestyle was mainly urban.

Since 1988, the study of Matsuoka et al. [38] in the United States had noticed the decline in circulating levels of 25(OH)D among long-term sunscreen users. The use of sunscreens protects the skin from erythematous radiation and skin cancers, but at the same time, it prevents the synthesis of vitamin D3 [39]. In our study, 64.7% of participants who used sun screen regularly had vitamin D deficiency, compared to 37.8% of those who did not.

Several factors have been described as influen cing the circulating levels of vitamin D such age, sex, wearing of the veil, phototype, socio-economic level, rural or urban residence, anthropometric parameters and dietetics. Our study confirms only the harmful effect of veiling and sunscreen use on vitamin D levels.

The association between PTH and vitamin D may be an important determinant of bone remodeling. A negative and significant correlation was found between PTH and 25(OH)D concentrations in the present study. Individuals with low vitamin D concentrations were those who had higher values of PTH, while individuals with high values of vitamin D sho wed low values of PTH. Similar results were observed in healthy individuals in Australia and Riga, and a value of 38 ng/mL was suggested as sufficient to avoid an increase in PTH [40] [41]. Other studies reinforce the negative correlation between vitamin D and PTH [42] [43].

Although our study is the first, since 2005, to update the national data concerning the vitamin D status in a healthy adult Tunisian population, certain limits should be noticed. Indeed, the population studied is not representative since most participants were living in urban areas with an average socio-economic level. Random sampling at the national level is necessary to overcome selection biases and ensure the representativeness of the population studied. Moreover, the number of participants could be increased to improve the strength of statistical tests.

## Conclusion

In conclusion, given the importance of vitamin D for health and the very high prevalence of this hormone deficiency, an adapted health policy is essential. A more widespread vitamin D supplementation and food fortification, especially during the winter months seems to be necessary in Tunisia.

## Dodatak

### Conflict of interest statement

All the authors declare that they have no conflict of interest in this work.
